# HSV-2 gE2/gI2 are immune evasion molecules that bind IgG Fc to inhibit antibody-dependent cellular cytotoxicity

**DOI:** 10.3389/fimmu.2026.1766722

**Published:** 2026-03-03

**Authors:** Giulia Tebaldi, Kevin P. Egan, Lauren M. Hook, Tina M. Cairns, Tomas Bergstrom, Kerry S. Campbell, Gary H. Cohen, Harvey M. Friedman

**Affiliations:** 1 Infectious Disease Division, Perelman School of Medicine, University of Pennsylvania, Philadelphia, PA, United States; 2 RNA Institute, Perelman School of Medicine, University of Pennsylvania, Philadelphia, PA, United States; 3 Department of Basic and Translational Sciences, School of Dental Medicine, University of Pennsylvania, Philadelphia, PA, United States; 4 Department of Infectious Diseases, University of Gothenburg, Gothenburg, Sweden; 5 Institute for Cancer Research, Fox Chase Cancer Center, Temple Health, Philadelphia, PA, United States

**Keywords:** antibody-dependent cellular cytotoxicity (ADCC), glycoproteins E and I, HSV-2, immune evasion, virus Fc receptor

## Abstract

**Introduction:**

HSV-2 glycoproteins C, D, and E (gC2/gD2/gE2) are immunogens included in an experimental HSV-2 vaccine. We evaluated whether these antigens serve as targets for antibody-dependent cellular cytotoxicity (ADCC).

**Methods:**

We transiently transfected HEK cells with gC2/gD2/gE2 DNA, added HSV-2 seropositive human convalescent sera (HCS), and measured surface CD107a expression on human NK cells by flow cytometry.

**Results:**

We demonstrated that antibodies to gC2/gD2/gE2 mediate ADCC. HSV-2 gE and gI form a complex that binds IgG Fc. We next determined whether gE2/gI2 inhibits ADCC, a crucial function mediated by the IgG Fc, by comparing ADCC titers when HCS were added to cells transfected with gD2, gI2, and a gE2 mutant (gE2_MUT_) unable to bind IgG Fc or gD2, gI2, and gE2 wild-type (gE2_WT_). ADCC titers increased by 6.5-fold when cells were transfected with the gE2_MUT_ versus gE2_WT_ (P=0.01). We then spiked HCS with a gE2 mAb that blocks IgG Fc binding, or with a gE2 mAb that does not block IgG Fc binding. The blocking mAb significantly increased titers (P<0.0001), whereas a non-blocking gE2 mAb had no effect.

**Discussion:**

We conclude that antibodies to gC2/gD2/gE2 are targets of ADCC, that gE2/gI2 inhibits ADCC, and that an mAb that targets the gE2 IgG Fc binding domain can prevent this inhibition.

## Introduction

Genital herpes infection is a significant cause of morbidity worldwide. It can be fatal when transmitted to newborns (1–3). Genital herpes lesions are painful for some individuals and devastating for many others because of stigma, social isolation, and feelings of shame (4, 5). Moreover, genital herpes increases the risk of HIV acquisition and transmission (6–8). These observations support the need for an effective vaccine (9). Despite extensive research, a vaccine that prevents genital herpes or treats individuals already infected has been elusive. Contributing factors include the complex mechanisms HSV uses to evade host innate and acquired immunity, including vaccine-induced immunity (10).

HSV-1 glycoprotein C (gC1) and HSV-2 glycoprotein C (gC2) are immune evasion molecules that bind to complement component C3b, thereby inhibiting complement activation (11–15). HSV-1 glycoprotein E (gE1) is also an immune evasion molecule that binds the Fc domain of IgG (16, 17). The binding domains for IgG Fc are located on gE1; however, HSV-1 glycoprotein I (gI1) forms a heterodimer with gE1, and the gE1/gI1 complex binds IgG Fc at a higher affinity (18–23). Notably, the HSV-1 FcR also binds the Fc domain of the IgG antibody targeting the virus, whereby the Fv domains of the antibody bind to the target antigen, and the Fc domain of the same antibody molecule binds to gE1 via a process we termed antibody bipolar bridging (17). We demonstrated that the HSV-1 FcγR protects infected cells from antibody-dependent cellular cytotoxicity (ADCC) and antibody-mediated complement activation *in vitro* and *in vivo* using a murine infection model (24–26). HSV-2 glycoprotein E (gE2) and glycoprotein I (gI2) also form an IgG FcγR (27, 28). However, differences have emerged between HSV-1 and HSV-2: gE1 alone can bind the IgG Fc domain, whereas gE2 requires gI2 for this activity (28). The goals of the current study were: i) to determine whether gC2, gD2, and/or gE2 are ADCC targets; ii) to assess whether gE2, as a complex with gI2, functions as an immune evasion molecule by preventing ADCC; and iii) to evaluate whether gE2/gI2 immune evasion can be blocked using mAbs directed against the IgG Fc binding domain on gE2.

## Materials and methods

### Sex as a biological variable

We do not know the sex of the 10 individuals who were the source of the human convalescent sera used in this study, as that information was de-identified.

### Cells

HEK293T cells (ATCC: CRL-11268) were cultured in α-MEM (Gibco, cat. 12561-056), containing L-glutamine, supplemented with 5% heat-inactivated fetal bovine serum (FBS; Gibco) and 1% penicillin/streptomycin (Gibco, cat. 15140-122).

NK-92 cell line (GFP-CD16–176 V/V) is an NK cell line engineered to express the high-affinity (176V/V; GenBank: BC017865.1) FcγRIIIA (CD16A). The cell line was purchased from ATCC (item number PTA-8836) and cultured in α-MEM complete media (Gibco, cat. 12571-063) supplemented with 10% heat-inactivated horse serum (Thermo Fisher, cat. 16050122), 10% FBS, and 1% penicillin/streptomycin. The cells were passaged every 4 days in the presence of hIL-2 (Corning, cat. F354043, lot 2283004), 200 U/mL, or clarified (filtered) J558L supernatant, which served as a source of human IL-2 (29, 30).

Human IL-2-producing J558L cells are derived from a mouse myeloma cell line that has been transfected with the huIL-2 cDNA, resulting in the stable release of human IL-2 into the supernatant (31). The cells were cultured in RPMI media (Gibco, cat. 75-085) containing L-glutamine and supplemented with 10% FBS, 1% penicillin/streptomycin, 1% α-MEM, 1% β-mercaptoethanol, and 1% HEPES 1M pH 7.4 (cat. 25060CI, ThermoFisher Scientific, Waltham, MA). Hu-IL-2-producing J558L cells produced human IL-2 that served as a growth supplement for NK-92 GFP-CD16 176V cells. Cells were allowed to grow for about 2 weeks, until the media turned yellow. The culture was centrifuged for 3 minutes at 395 × g, the supernatant was filtered through a 0.22 μm filter, aliquoted, and frozen at -80°C (32).

SKOV3 cells are human ovarian cancer cells that express the HER2 receptor, making them suitable for NK cell ADCC studies when a therapeutic human IgG1 antibody to HER2 (Trastuzumab/Herceptin) is added. These cells serve as a positive control for CD107a surface expression on NK cells. SKOV3 cells were cultured in RPMI media containing L-Glutamine and supplemented with 5% FBS and 1% penicillin/streptomycin.

All cells were maintained in 5% CO_2_ at 37 °C.

### Plasmids

Protein sequences of the full-length gC2 and gD2 were derived from HSV-2 strain 333, while the full-length sequences of gE2 and gI2 were derived from HSV-2 strain 2.12 (GenBank: AQZ56445.1 for gC2; AMB66102.1 for gD2; for gE2 and gI2, the sequences and locations of the gE2 mutations are described in **Supplementary Figure 1**) (21, 28). The DNA sequences were cloned into the pcDNA3.1(+) vector, which is codon-optimized for expression in human cells, and sequenced to verify accuracy. The size was also checked by agarose gel electrophoresis at GenScript (Piscataway, NJ, USA).

### Transfection

Sub-confluent HEK293 cells were transiently transfected with gC2, gD2, and gE2, or gD2, gE2, and gI2 plasmids using 22.5 μg of total DNA (7.5 μg for each plasmid) mixed with 56.5 μg PEI (1mg/mL) (PEI transfection reagent, Polysciences, cat. 26008) in 1.5 mL of Dulbecco’s modified essential medium (DMEM) (Gibco, cat. 11965-084) without FBS. After 15 min at R.T., 6 mL of DMEM was added, and the transfection solution was incubated overnight at 37 °C in 5% CO2 on the HEK293 cell monolayer. We used DL6 mAb to detect gD2, mAb B1E6 and mAb E1 for gE2, H1196 (purchased from Virusys, now SeraCare, cat. P1124) for gC2, and rabbit polyclonal Ab UP1928 for gI2 (33–35).

### Human pooled IgG and HSV-2 human convalescent sera

Ten HCS served as the source of antibodies for the NK cell CD107a surface expression assays. The HCS were provided by Anna Wald and Christine Johnston and were obtained from subjects with genital herpes (**Table 1**). The individual HSC and the pooled human IgG (IgG from human sera, cat. 56834, Sigma) were pre-adsorbed with the HEK293 cells for 1 to 2 hours at R.T. The sera were centrifuged twice for 10 min at 9,500 x g, the supernatant removed, aliquoted, and stored at -20°C.

**Table 1:** Serum IgG ELISA and ADCC endpoint titers of individual HSV-2 HCS.

Sample ID	Annual genital recurrences	Years post infection	Serum IgG ELISA endpoint titer			ADCC endpoint titer*
			gC2	gD2	gE2	
#385	0	unknown	1:64,000	1:64,000	1:16,000	1:3,200
#365	6	4	1:16,000	1:32,000	1:64,000	1:3,200
#416	6	3	1:32,000	1:16,000	1:16,000	1:3,200
#487	6	11	1:32,000	1:32,000	1:32,000	1:6,400
#540	6	14	1:32,000	1:128,000	1:16,000	1:6,400
#371	7	14	1:16,000	1:64,000	1:32,000	1:6,400
#392	10	9	1:16,000	1:128,000	1:32,000	1:12,800
#348	12	17	1:64,000	1:64,000	1:64,000	1:6,400
#353	12	10	1:256,000	1:512,000	1:64,000	1:3,200
#386	14	2	1:128,000	1:32,000	1:64,000	1:3,200
Pooled human IgG	Unknown	Unknown	1:64,000	1:128,000	1:64,000	1:6,400

*If an endpoint titer was not reached at the 1:3,200 dilution, ADCC endpoint titers were calculated by regression analysis and adjusted to the closest lower two-fold dilution number.

### ELISA assays

ELISA was performed to measure antibodies to gC2, gD2, and gE2 in the HCS. 100 ng of gC2, gD2, or gE2 was used, and the assay was performed as previously described (36).

### NK cell CD107a surface expression assay by flow cytometry

HEK293 cells were transiently transfected to express gC2, gD2, and gE2, or gD2, gE2, and gI2. Twenty-four hours later, these cells served as target cells for NK cell activation (effector cells). The following day, the transfection media was removed, the target cells were washed at R.T. with PBS, detached using 0.05% trypsin-EDTA at 37°C, washed, and viable cells counted by trypan blue exclusion. 3x10^5^ target cells were incubated with pre-adsorbed pooled human IgG or HCS for 30 min. at R.T. NK effector cells were washed to remove hu-IL-2, viable cells counted using trypan blue, and 3x10^5^ cells were added to the target cells at a 1:1 ratio for two hours at 37°C in the presence of a 1:1000 dilution of monensin (BioLegend, cat. 420701), and 200 ng of anti-CD107a (LAMP1, clone H4A3, cat. 328619, BioLegend). The cells were washed twice with PBS, and Live/Dead IR stain was added for 10 min at R.T. in the dark, followed by three PBS washes, then fixed with 1% paraformaldehyde in PBS and stored at 4 °C until flow cytometry analysis the next day. Flow cytometry data were collected using a BD FACSymphony A5 SE and analyzed with FlowJo v10.8.1. Fifty thousand events were collected per sample, gated on singlets, and each condition was performed in duplicate.

The following reagents were used for flow cytometry. Monensin solution (cat. 420701, BioLegend, used at 1:1000); PMA (phorbol 12-myristate 13-acetate) cell activation cocktail (without Brefeldin A), composed of PMA and ionomycin (cat. 423301, BioLegend, used at 0.5 μl/well in a final volume of 200 μl); APC anti-human CD107a (LAMP-1); live/dead™ fixable near-ir dead dell stain kit (cat. L10119, Invitrogen); Alexa Fluor 488 anti-mouse IgG2A (Clone RMG2a-62, cat. 407121, BioLegend); Alexa Fluor 488 goat anti-mouse IgG (Clone Poly4053, cat. 405319, BioLegend); Alexa Fluor 488 goat anti-rabbit IgG (H+L) (A11034, LifeTechnology); mouse anti-human IgG Fab PE (cat. MA1 = 10377, Invitrogen). Herceptin (Trastuzumab) was obtained from the pharmacy at Fox Chase Cancer Center. A 10% aqueous paraformaldehyde solution (EM grade) was diluted to 1% in PBS (cat. 15712, Electron Microscopy Science).

### Biosensor assay

HSV-1 and HSV-2 gE and gE/gI heterodimers were purified in our lab using baculovirus-produced proteins, as previously described (36). The gE1 and gE2 proteins were purified on a Ni column, while the gE1/gI1 and gE2/gI2 heterodimers were purified on a Ni column followed by an anti-FLAG column. Purification was facilitated by C-terminal His tags on gE1 and gE2, or by FLAG tags on gI1 and gI2. We evaluated whether human IgG Fc binds to gE1, gE2, gE1/gI1, or gE2/gI2 using SPR (Biacore 3000). Equivalent amounts (~110 RU) of either gE1, gE2, gE1/gI1, or gE2/gI2 were captured to an anti-HIS coupled CM5 chip via binding of their C-terminal His tags. Next, 750 nM of non-immune human IgG was injected across the chip surface for 120-240s. The RU from a control flow cell where only non-immune human IgG was injected (no gE) was subtracted from each curve.

Biosensor assays were also used to assess whether mAbs block human IgG Fc binding to gE2/gI2. Purified gE2/gI2 heterodimer (~200 RU) was captured on a CM5 anti-His chip via a C-terminal His-tag on gE2 with a flow rate of 10 µl/min in HBS-EP+ (10 mM HEPES, 150 mM NaCl, three mM EDTA, pH 7.4) using a Biacore 1k+ instrument (Cytiva). Murine non-immune IgG (IgG from mouse serum I5381, Sigma Aldrich) and mAbs E1 and B1E6 at concentrations of 200 µg/ml, flow rate 20 µl/min in HBS-EP+ or HBS-EP+ alone, were then injected across separate flow cells for 120 sec over the captured gE2/gI2 heterodimer. Human IgG1 isotype (InVivoMab, VWR) at a concentration of 400 µg/mL, with a flow rate of 10 µL/min in HBS-EP+, was then injected over all flow cells for 60 seconds. The RU from a control flow cell where only human IgG1 was injected (no gE2/gI2, no Abs/mAbs) was subtracted from each curve. A 100 mM glycine solution, pH 2.0, was used to regenerate the CM5 anti-His chip to baseline levels. The % blocking of each antibody was calculated near the end of the human IgG1 injection (~60 sec) using the following equation: % blocking = 100 - [(RUTest/RUBuffer alone)*100].

### Statistical analysis

Analyses were done using GraphPad Prism 10. Details of the statistical method used are included in the text when P values are noted, or in the figure legends. P values <0.05 were considered statistically significant.

### Study approval

The use of de-identified serum samples provided by colleagues from another USA institution was considered Exempt by the University of Pennsylvania IRB.

## Results

### CD107a surface expression as a marker of NK cell activation

We developed an ADCC assay to measure antibodies in human sera that target immunogens included in a trivalent gC2/gD2/gE2 mRNA genital herpes vaccine. BioNTech SE has developed a modified version of this trivalent vaccine, which is currently in human trials (ClinicalTrials.gov; NCT05432583) (36). ADCC assays require target cells expressing surface antigens. IgG antibodies bind to the surface antigens by their Fv domains, and their Fc domains bind to IgG Fc receptors (FcγR) on the NK effector cells.

**Figure 1:**
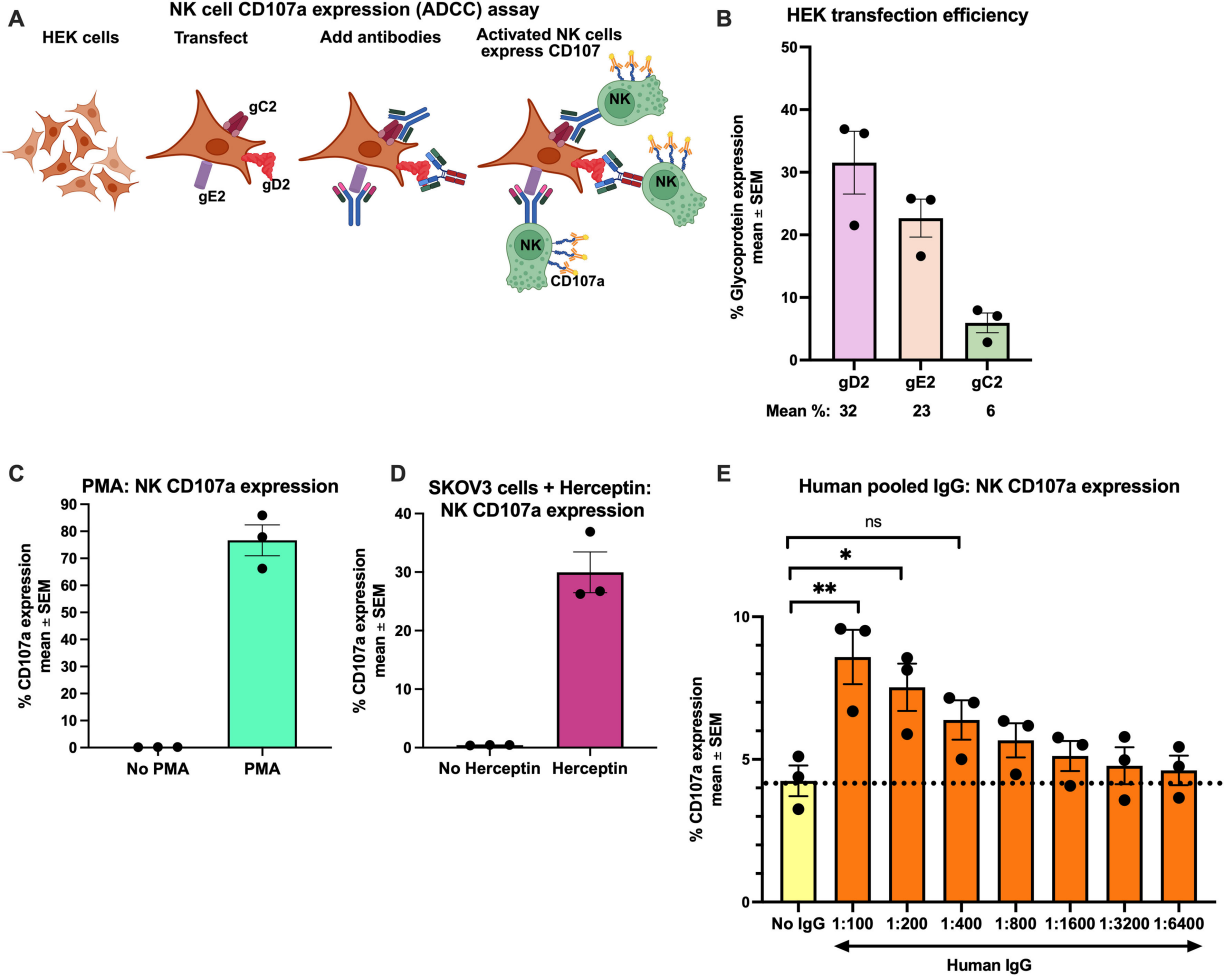
Establishing the CD107a NK cell (ADCC) assay with HSV-2 gC2, gD2, and gE2 as targets. Schematic representation of the assay to measure NK cell CD107a expression. HEK293 cells are transfected with three separate plasmids expressing full-length gC2, gD2, or gE2. Twenty-four hours later, the cells are incubated with pooled human IgG for 30 minutes at room temperature. Then, NK cells are added for 2 hours, and NK cell CD107a expression is determined by flow cytometry **(A)**. Glycoprotein expression. The mean percent of cells expressing gC2, gD2, and gE2 (n=3) **(B)**. Positive control for NK cell activation using PMA **(C)**. Positive control for NK cell CD107a expression using Herceptin (Trastuzumab) antibody to HER2 and SKOV3 cells that express high levels of HER2 **(D)**. NK cell CD107a expression using pooled human IgG as the antibody source. HEK293 cells were transfected with gC2, gD2, and gE2 DNA. Serial dilutions of pooled human IgG were added (1:100 to 1:6400), followed by NK cells (n = 3). **P<0.01, *P<0.05, ns not significant. The dotted line indicates ADCC endpoint titers based on the % NK cell CD107a expression when no IgG was added (negative control). P values were calculated by Ordinary one-way ANOVA adjusted for multiple comparisons. *P<0.05; **P<0.01; ns, not significant **(E)**.

We used HEK293 cells transiently transfected with HSV-2 DNA expressing full-length gC2, gD2, and gE2 as target cells. The antibody source was either human convalescent serum (HCS) obtained from HSV-2 seropositive subjects with 0–14 annual recurrences of genital herpes (provided by Anna Wald and Christine Johnston), or human IgG, pooled and purified from serum of multiple donors (Sigma). As effector cells, we used a human NK cell line, NK-92, which expresses the high-affinity homozygous (176V/V) IgG FcγRIIIA (CD16A) (29, 30). These cells were chosen because in humans, NK cells that express FcγRIIIA are the effector cells that most commonly mediate ADCC (37–39). Additionally, by using an NK cell line, we avoided donor-to-donor variability that can occur with NK cells from multiple donors. We used flow cytometry to quantify surface expression of CD107a, a marker of NK cell activation (**Figure 1A**) (40–42).

First, we evaluated the efficiency of HEK293 cell transfection by gC2, gD2, and gE2 DNA. The three plasmids expressing full-length glycoproteins were added in equal quantities, and surface expression of the glycoproteins was evaluated by flow cytometry. Expression of the three glycoproteins was unequal, with 32% of cells expressing gD2, 23% gE2, and 6% gC2 (ratio 5:4:1 of gD2:gE2:gC2) (**Figure 1B**). For this reason, we assessed the expression levels for transfected HEK cells in each series of experiments in this study. However, this ratio is comparable to the expression of these glycoproteins in infected cells (43). We chose to use transfected cells as ADCC targets rather than infected cells because we could control cell viability more effectively after transfection than after infection. In addition, transfected cells allow us to determine whether gC2, gD2, and gE2 are targets of ADCC in HCS, a question that cannot be addressed with infected cells. We used two positive controls for activating NK cells. One involved stimulating NK cells with phorbol 12-myristate 13-acetate (PMA), a potent inducer of surface CD107a expression on NK cells (**Figure 1C**), while the other used SKOV3 cells that constitutively express HER2 receptors that serve as a target for Trastuzumab (Herceptin) antibody, followed by adding NK-92 cells (**Figure 1D**) (44). Both controls demonstrated robust expression of CD107a on NK cells. To measure ADCC, we initially used human IgG pooled from multiple donors as the source of antibody. As a negative control for ADCC, we incubated gC2/gD2/gE2-transfected HEK cells with NK cells in the absence of antibody (**Figure 1E**, No IgG). The negative control was then used to determine an endpoint titer for the pooled human IgG, which was ≥ 1:6400 (**Figure 1E**, dotted line). We conclude that this assay is effective for measuring NK cell activation, and that gC2, gD2, and/or gE2 are ADCC targets for antibodies in pooled human sera.

### NK CD107a surface expression using HSV-2 HCS as the antibody source and gC2/gD2/gE2 as targets

**Figure 2:**
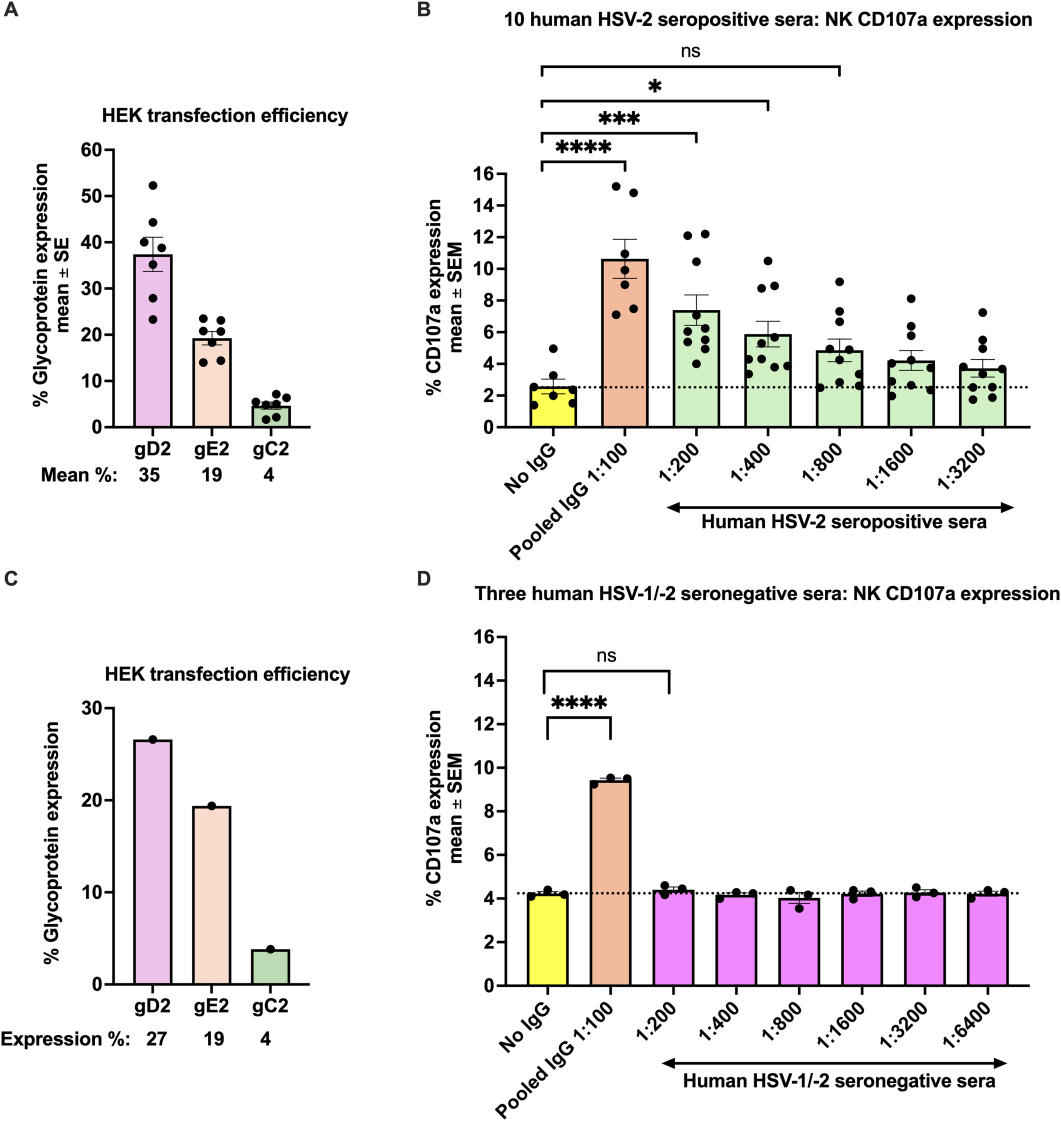
NK cell CD107a expression using seropositive HCS or seronegative sera. Transfection efficiency of the 10 seropositive HCS **(A)** or three HSV-1/-2 seronegative sera **(C)**. No IgG serves as a negative control for NK cell CD107a expression; pooled human IgG at a 1:100 dilution is used as a positive control. The means for 10 seropositive HCS are shown for dilutions ranging from 1:200 to 1:3200 **(B)**, or for three HSV-1/-2 seronegative sera at dilutions ranging from 1:200 to 1:6400 **(D)**. In **(B, D)**, ****P<0.0001, ***P<0.001, *P<0.05, ns not significant. P values were calculated by the Ordinary one-way ANOVA adjusted for multiple comparisons. The dotted line indicates ADCC endpoint titers based on the % NK cell CD107a expression when no IgG was added (negative control).

We next evaluated NK cell CD107a expression using 10 sera obtained from HSV-2-infected individuals as the source of antibody. The transfection efficiency was determined (**Figure 2A**). It varied little from the prior experiment (**Figure 1B**). The positive controls for NK cell activation, using PMA and SKOV3 cells (**Supplementary Table 1**), also varied little from those shown in **Figures 1C, D**. The negative control had gC2, gD2, gE2-transfected HEK cells plus NK-92 cells with no antibody. In contrast, the positive control used pooled human IgG at 1:100 dilution (**Figure 2B**). The 10 human convalescent sera were evaluated at dilutions ranging from 1:200 to 1:3200. The endpoint titer for the 10 HCS was ≥1:3200 (**Figure 2B**). We conclude that HCS contains high titers of antibodies to one or more of the vaccine antigens, gC2, gD2, and gE2, and that target cells coated with these antibodies induce CD107a expression on NK cells.

As an additional negative control, we evaluated CD107a expression using three HSV-1/-2 double seronegative human sera as a source of antibodies. We expected that serial dilutions (1:200 to 1:6400) of the seronegative sera would not stimulate NK cell CD107a expression beyond background levels noted when NK cells were exposed to transfected cells without antibody. The transfection efficiency for gC2/gD2/gE2 showed the same general pattern as in prior experiments (**Figure 2C**), as did the controls for NK cell CD107a expression (**Supplementary Table 1**). As expected, the human seronegative sera did not induce NK cell CD107a expression above background levels of the negative control (**Figure 2D**, No IgG). We conclude that HSV-2 seropositive HCS stimulates NK cell CD107a expression, while seronegative serum does not.

We compiled the results of the 10 HCS samples and the pooled human IgG to display the number of annual genital recurrences, years since the onset of genital disease, gC2, gD2, and gE2 IgG binding (ELISA) endpoint titers, as well as ADCC endpoint titers, as determined by NK cell CD107a expression (**Table 1**). All 10 subjects produced IgG binding antibodies to gC2, gD2, and gE2, and all had ADCC titers ranging from 1:3,200 to 1:12,800. We did not detect a correlation between the number of genital recurrences per year and ADCC endpoint titers (r = 0.1144, P value not significant); however, only one of the 10 HCS samples was from a subject with fewer than six annual recurrences. Perhaps the narrow range of recurrent genital infection episodes accounts for the lack of correlation. We conclude that gC2, gD2, and/or gE2 serve as targets of ADCC.

### HSV-2 gE2 and gI2 are required for IgG Fc binding

**Figure 3:**
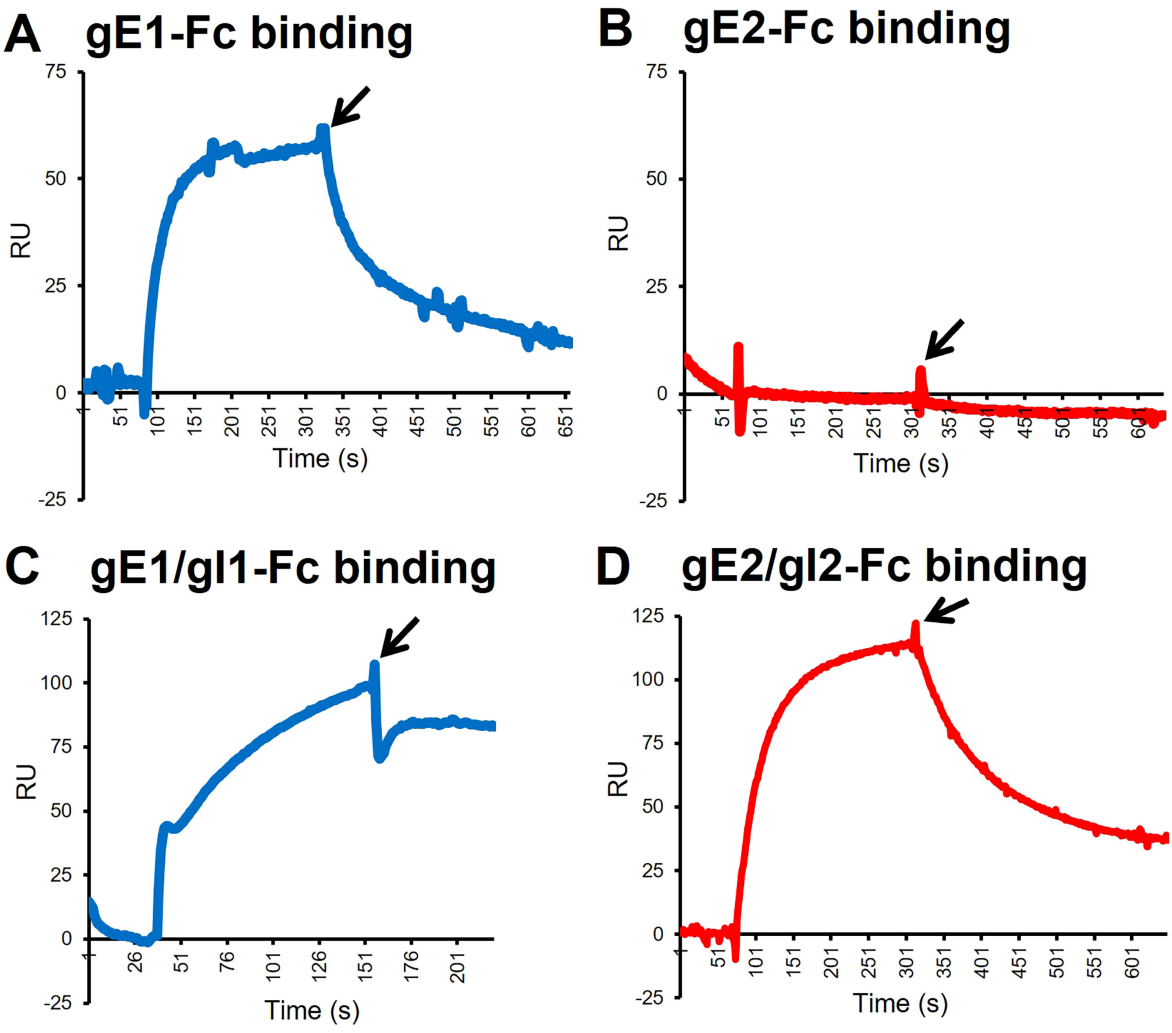
SPR graphs demonstrating binding of HSV-1/2 seronegative human IgG Fc. HSV-1 gE1, gE1/gI1, or HSV-2 gE2, gE2/gI2 were bound to the chip, and HSV-1/2 double seronegative (non-immune) human IgG was flowed over the chip. IgG Fc binding to gE1 **(A)**. IgG Fc binding to gE2 **(B)**. IgG Fc binding to gE1/gI1 **(C)**. IgG Fc binding to gE2/gI2 **(D)**. Arrows indicate the time when the flow of non-immune human IgG was discontinued.

Prior reports indicated that HSV-2 gE2 differs from HSV-1 gE1 in that gE1 binds human IgG Fc as a monomer or as a heterodimer with HSV-1 gI1. In contrast, HSV-2 gE2 only binds human IgG Fc as a heterodimer with HSV-2 gI2 (28). We used surface plasmon resonance (SPR) to confirm these observations. HSV gE1 or gE2 was captured on the SPR chip. Human IgG purified from an HSV-1/2 double-seronegative donor serum was then flowed over the chip. Human IgG bound to gE1 (**Figure 3A**) but not to gE2 (**Figure 3B**). Next, we captured the HSV-1 gE1/gI1 complex or the HSV-2 gE2/gI2 complex on the SPR chip and added human IgG. Both gE1/gI1 and gE2/gI2 bound non-immune human IgG, although the kinetics of binding differed, with gE2/gI2 showing faster on and off rates than gE1/gI1 (**Figures 3C, D**). These results confirm those of Galli et al. and indicate that to assess the importance of gE2 in immune evasion, cells need to be transfected with both gE2 and gI2, as performed in the ensuing experiments (28).

### HSV-2 gE2 mutants defective in IgG Fc binding

**Figure 4:**
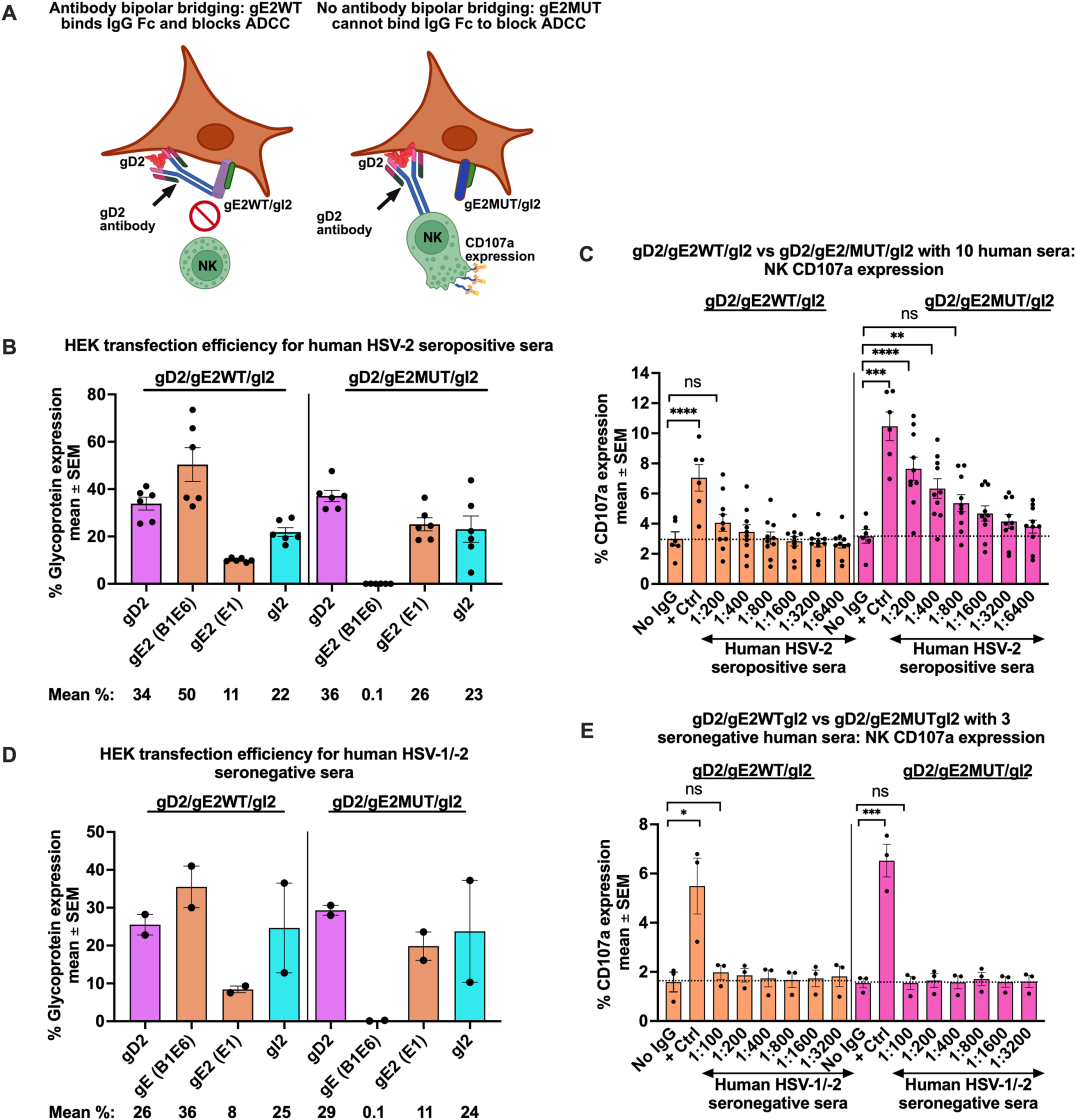
HSV-2 gE2 inhibits NK cell CD107a expression. Experimental model: Left side: Full-length gD2, gE2, and gI2 proteins are expressed at the surface of HEK target cells. Pooled human IgG is added as a source of antibodies, and activation of NK effector cells is measured by flow cytometry. The model depicts antibody bipolar bridging, which prevents NK cells from accessing the IgG Fc domain, resulting in minimal CD107a expression by NK cells. Right side: A mutation in the gE2 IgG Fc binding domain prevents antibody bipolar bridging, resulting in higher levels of NK cell CD107a expression. **(A)** Transfection efficiency for experiments shown in **Figure 4C**. B1E6 identifies gE2_WT_ but not gE2_MUT#2_, E1 identifies both gE2_WT_ and gE2_MUT#2_. **(B)** NK cell CD107a expression when human HSV-2 HCS is added as antibody source (n=10 sera). **(C)** Transfection efficiency for experiments shown in **Figure 4E**. **(D)** NK cell CD107a expression when human HSV-1/2 seronegative sera are added as the antibody source for NK cell activation (n=3 sera). **(E)** P values were calculated by the Ordinary one-way ANOVA adjusted for multiple comparisons. *P<0.05; **P<0.01; ***P<0.001; ****P<0.0001; ns, not significant.

One of our goals was to determine whether gE2/gI2 inhibits ADCC by binding the IgG Fc domain of antibodies that target HSV-2 antigens expressed on transfected cells. We postulated that wild-type gE2/gI2 (gE2_WT_) would reduce NK cell CD107a expression compared with gE2 mutant (gE2_MUT_)/gI2 because of antibody bipolar bridging, where gE2_WT_ binds the IgG Fc domain of the antibody that is bound to HSV-2 antigens by the IgG Fv domain (antibody bipolar bridging) (**Figure 4A**) (17). We prepared three gE2 mutants, two of which were based on published reports describing gE2 mutations that disrupt IgG Fc binding to gE2/gI2, and one gE2 mutation was at a location homologous to a site in HSV-1 gE1 that abolished IgG Fc binding (28, 45) (**Supplementary Figures 1A, B**). We confirmed that all three mutants were expressed at the cell surface when co-transfected with gD2 and gI2 (**Supplementary Figure 2A**), and that the three gE2 mutants failed to bind human non-immune IgG Fc (**Supplementary Figure 2B**). We selected gE2_MUT#2_ for further studies of NK cell CD107a expression.

### HSV-2 gE2/gI2 inhibits ADCC

HEK cells were transfected with gD2, gI2, and gE2_WT_ or gE2_MUT#2_ DNA. We omitted gC2 DNA due to difficulties in achieving good surface expression of gC2 when used in combination with the other three DNA plasmids. Transfection efficiency was assessed using antibodies to detect gD2, gI2, and gE2_WT_ or gE2_MUT#2_ expression. Two different mouse monoclonal Abs (mAbs) were used for gE2 detection: B1E6, which targets the Fc binding domain, and E1, which recognizes an epitope on gE2 not involved in IgG Fc binding. The binding of either mouse mAb to gE2 reflects binding by their IgG Fv domain because the IgG Fc domain of mouse IgG does not bind to gE or gE/gI of HSV-1 or HSV-2 (28, 46). Both mAbs recognized gE2_WT,_ but only E1 bound to gE2_MUT#2_ (**Figure 4B**). The same 10 HSV-2 HCS as in **Figure 2** were used to evaluate gE2/gI2 inhibition of NK cell CD107a expression by comparing ADCC endpoint titers when gD2/gE2_WT_/gI2 was the target cell compared with gD2/gE2_MUT_/gI2. NK cell CD107a expression was higher in cells transfected with the gE2_MUT#2_ than gE2_WT_ (**Figure 4C**, **Table 2**). The geometric mean endpoint ADCC titer was 1:985 for cells transfected with gE2_WT,_ compared to 1:6400 for cells transfected with gE2_MUT#2_, representing a 6.5-fold difference (P<0.01, by two-tailed Mann-Whitney test). This increase in titer occurred despite high titers of gE2/gI2 IgG binding antibodies (as measured by ELISA) in the HCS (**Table 2**). This result suggests that gE2/gI2 antibodies produced by infection do not prevent gE2_WT_/gI2 from inhibiting ADCC.

**Table 2:** HSV-2 HCS ADCC endpoint titers for gD2/gE2_WT_/gI2 and gD2/gE2_MUT_/gI2.

Sample ID	gE2/gI2 IgG ELISA endpoint titer	ADCC endpoint titer for gD2/gE2_WT_/gI2	ADCC endpoint titer for gD2/gE2_MUT_/gI2*
#385	1:32,000	1:200	1:1,600
#365	1:32,000	1:6,400	1:12,800
#416	1:16,000	1:1,600	1:6,400
#487	1:64,000	1:800	1:6,400
#540	1:32,000	1:400	1:6,400
#371	1:32,000	1:200	1:3,200
#392	1:64,000	1:200	1:6,400
#348	1:32,000	1:1,600	1:6,400
#353	1:64,000	1:3,200	1:12,800
#386	1:32,000	1:6,400	1:12,800
Geometric mean endpoint titer	1:36,758	1:985	1:6,400

* If an endpoint titer was not reached at the 1:6,400 dilution, ADCC endpoint titers were calculated by regression analysis and adjusted to the closest lower two-fold dilution number.

We evaluated NK cell CD107a expression using HSV-1/2 double-seronegative human sera as the source of antibody, with the expectation that the seronegative human sera would not stimulate NK cell CD107a expression. We confirmed the transfection efficiency (**Figure 4D**). As expected, the seronegative human sera did not trigger expression of NK cell CD107a using either gD2/gE2_WT_/gI2, or gD2/gE2_MUT#2_/gI2 transfected cells as targets (**Figure 4E**). We conclude that a mutation that abrogates explicitly IgG Fc binding leads to higher NK cell CD107a expression, supporting our hypothesis that gE2_WT_/gI2 inhibits ADCC.

### mAb B1E6 blocks IgG Fc binding to gE2_WT_, preventing gE_WT_/gI2 from inhibiting ADCC

Another goal was to determine whether we can prevent gE2_WT_/gI2 from inhibiting ADCC by using an antibody that targets the IgG Fc binding domain on gE2 to block its immune evasion activity. We reasoned that a positive result would support efforts to produce blocking antibodies by gE2 immunization. These experiments required two gE2 mAbs with differing properties, one that blocks IgG Fc binding to gE2 and another that does not. We postulated that the mAb that binds to gE2 and blocks IgG Fc binding would enhance the level of ADCC, while the mAb that binds gE2 but does not block would have no effect.

**Figure 5:**
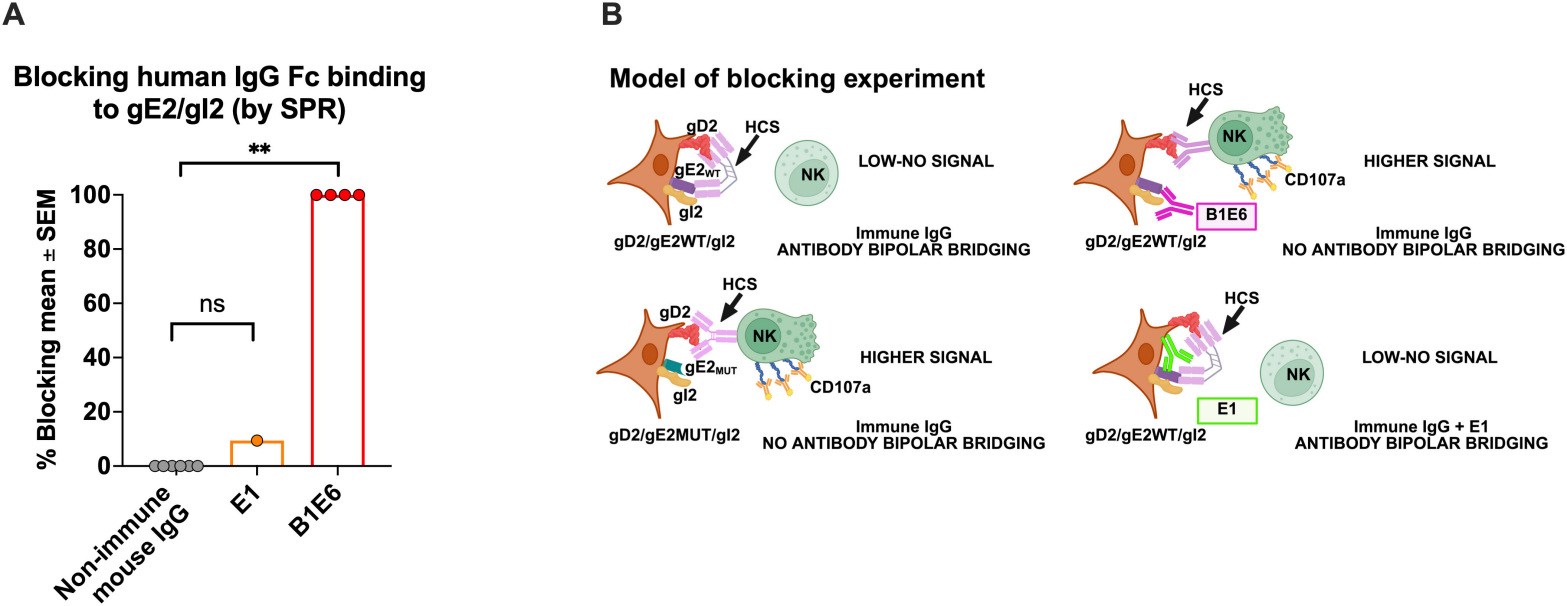
Identifying a gE2 mAb that blocks IgG Fc binding to gE2/gI2. The gE2/gI2 complex was added to a chip. Non-immune murine IgG, mAb E1, or mAb B1E6 was then flowed over the chip, followed by the addition of human non-immune IgG. B1E6, but not E1 or non-immune mouse IgG, blocked IgG Fc binding of human non-immune IgG to gE2/gI2. **(A)** Cartoon model of adding B1E6 or E1 to block human IgG Fc binding to gE2/gI2. HEK cells are transfected with gE2_WT_ or gE2_MUT_ DNA. Antibody bipolar bridging develops when HCS is added to cells transfected with gD2/gE2_WT_/gI2 DNA (upper left model), but not when transfected with gD2/gE2_MUT_/gI2 DNA (lower left model). B1E6 prevents IgG Fc binding to gE2, thereby increasing NK cell CD107a expression by HCS (upper right model), while E1 does not block IgG Fc binding and has no effect on NK cell CD107a expression (lower right model). **(B)** P values were calculated using Dunn’s multiple comparisons test. **P<0.01; ns, not significant.

We chose mAb B1E6 based on it not binding to gE2_MUT#2_, suggesting that B1E6 recognizes an epitope involved in IgG Fc binding. A prior publication noted that the B1E6 epitope was detected in all but 2 of 2,400 HSV-2 clinical isolates, indicating that this epitope is highly conserved (47). We chose mAb E1 based on it binding to each of the gE2_MUTs,_ indicating that E1 does not interact with any of the epitopes modified by these mutations (**Figure 4B**; **Supplementary Figure 2A**). As a further approach, we evaluated whether these mAbs block IgG Fc binding to gE2/gI2 in a biosensor (surface plasmon resonance) assay. The gE2/gI2 complex was added to a nickel-coated chip via a gE2 His-tag, followed by the flow of non-immune mouse IgG, mAb B1E6, or mAb E1 over the chip, and then non-immune human IgG. B1E6 totally blocked non-immune human IgG Fc binding to gE2_MUT#2_/gI2, while E1 and non-immune mouse IgG had little or no effect, respectively (**Figure 5A**; **Supplementary Figures 2A, B**). B1E6 and E1 meet our goal of having one mAb that blocks human IgG Fc binding and another, as a control, that fails to block (experimental model, **Figure 5B**).

**Figure 6:**
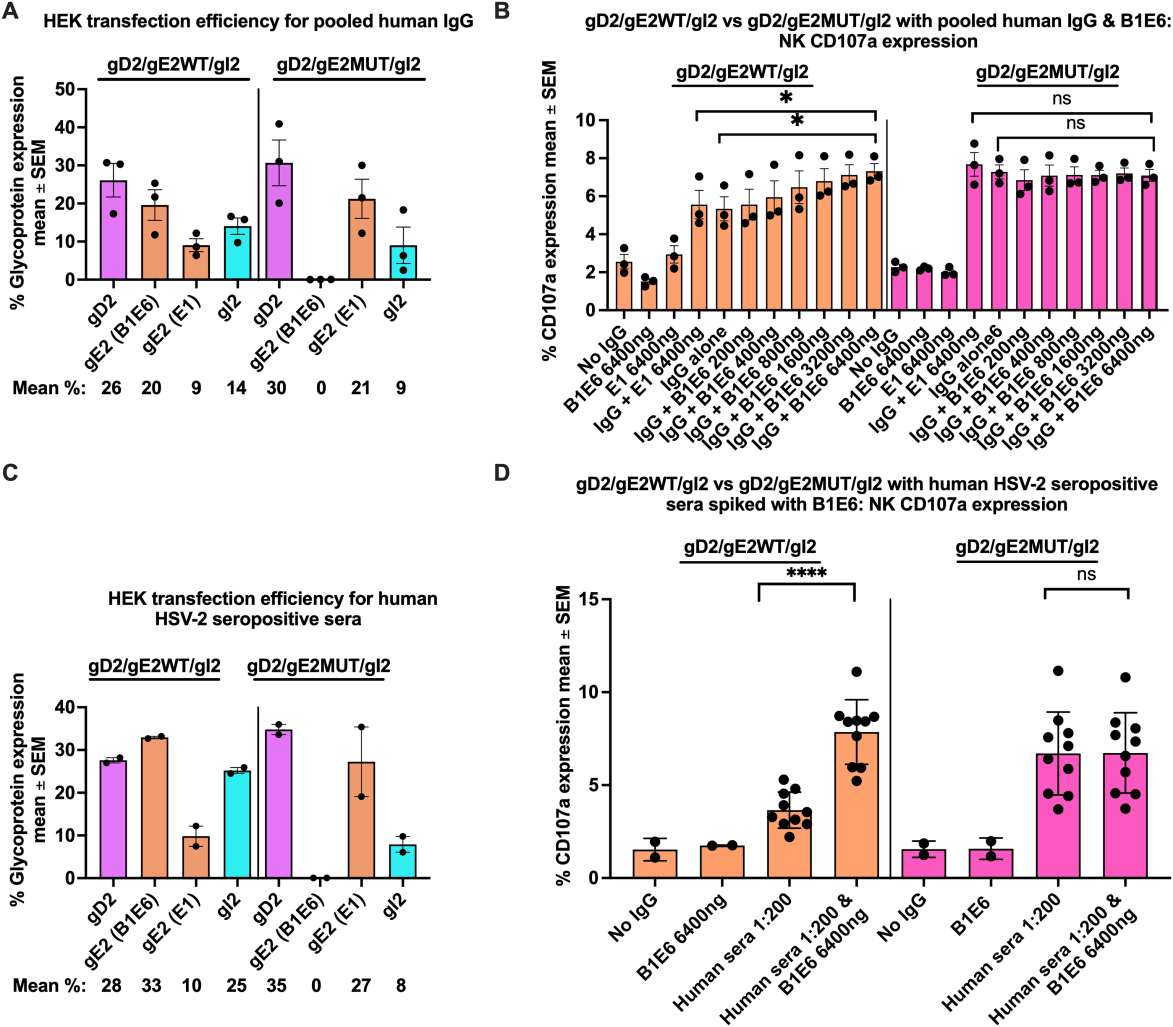
Spiking human sera with B1E6, a gE2 mAb that blocks IgG Fc binding, or E1, a gE2 mAb that does not block IgG Fc binding. Transfection efficiency in HEK cells used for experiments in **(B)**. **(A)** Spike experiment; E1 at 6400ng or B1E6 at concentrations ranging from 200 to 6400ng was added to the pooled human IgG (1:100 dilution). B1E6, but not E1, increases NK cell CD107a expression of cells transfected with gE2_WT_, but not gE2_MUT_, DNA **(B)**. Transfection efficiency in HEK cells used for experiments in **(D)**. **(C)** B1E6 increases NK cell CD107a expression of cells transfected with gE2_WT_, but not gE2_MUT_, using 10 HCS sera **(D)**. P values in **(B)** were calculated by paired t-test, and in **(D)** by the two-tailed Mann-Whitney test. *P<0.05; ****P<0.0001; ns, not significant.

We transfected HEK cells with gD2, gI2, and gE2_WT_ or gE2_MUT#2_ and confirmed transfection efficiency (**Figure 6A**). B1E6 and E1 both bound to gE2_WT_, while only E1 bound to gE2_MUT#2_ (**Figure 6A**). Next, we spiked pooled human IgG with B1E6 or E1 (**Figure 6B**). We hypothesized that B1E6 would block IgG Fc binding to gE2_WT_ but not gE2_MUT#2_ and would increase NK cell CD107a expression of cells transfected with gE2_WT_ but not gE2_MUT#2_. In addition, we postulated that mAb E1 would not affect NK cell CD107a expression in cells transfected with gE2_WT_ or gE2_MUT#2_. The first three sets of bars in **Figure 6B** (both left and right panels) are controls: going from left to right, the first set of bars (labeled No IgG) shows NK cell CD107a expression when HEK cells are transfected with gD2/gE2_WT_/gI2, or gD2/gE2_MUT#2_/gI2 in the absence of antibody. These controls establish the background NK cell CD107a expression in the absence of antibody. The second and third sets of bars (labeled B1E6 6400ng and E1 6400ng, respectively) show that mAbs B1E6 and E1, used at the highest concentration, do not induce NK cell CD107a expression, because mouse IgG Fc does not engage with the human FcγRIIIA on NK cells (48). The remaining sets of bars involve pooled human IgG as the source of antibodies. The first of these, labeled IgG + E1 6400ng, shows that mAb E1, even at the high concentration of 6400ng, when added to pooled human IgG, does not affect NK cell CD107a expression of cells transfected with gD2/gE2_WT_/gI2 or gD2/gE2_MUT#2_/gI2 (compare the fourth bar with the one immediately to the right labeled IgG alone). The remaining six bars show the result for NK cell CD107a expression when pooled human IgG is spiked with increasing concentrations of B1E6. As the B1E6 concentration increases from 200ng to 6400ng, NK cell CD107a expression increases for cells transfected with gD2/gE2_WT_/gI2, while B1E6 does not affect NK cell CD107a expression of cells transfected with gD2/gE2_MUT#2_/gI2 DNA. We conclude that gE2 mAb B1E6, which blocks IgG Fc binding, increases NK cell CD107a expression when added to pooled human IgG (P<0.05 compared to no B1E6), and that gE2 mAb E1, which does not block IgG Fc binding, does not increase NK cell CD107a expression. These results support the hypothesis that antibodies that target the Fc binding domain on gE2 can bind and block its immune evasion properties.

We next evaluated the effect of spiking each of the 10 HSV-2 HCS with B1E6, instead of pooled human IgG. Transfection efficiency of the HEK cells was confirmed (**Figure 6C**). As with pooled human IgG, B1E6 increased NK cell CD107a expression in cells transfected with gE2_WT_ DNA (P<0.0001) (**Figure 6D**, left panel). In contrast, B1E6 had no effect when cells were transfected with gE2_MUT#2_ DNA (**Figure 6D**, right panel). We conclude that gE2_WT_/gI2 blocks ADCC, and that mAb B1E6 can overcome this block to enhance ADCC responses. These 10 HCS have high titers of gE2/gI2 binding antibodies (**Table 2**). However, the HCS is only capable of preventing gE2/gI2 from inhibiting ADCC when B1E6 is added. These results indicate that blocking antibodies targeting the IgG Fc binding domains on gE2/gI2 are not present in HCS sera at sufficient titer to block IgG Fc binding, perhaps because the immune evasion domain(s) on gE2/gI2 are not very immunogenic.

## Discussion

We used HSV-2 seropositive serum to demonstrate that gC2, gD2, and/or gE2 expressed as full-length proteins at the surface of transfected HEK cells are targets for ADCC. Surface expression of CD107a on NK cells, as detected by flow cytometry, indicates NK cell activation and serves as a biomarker for ADCC (29, 30). We confirmed that co-expression of gE2 with gI2, but not gE2 alone, is required for IgG Fc binding, and that a gE2 mAb that targets the IgG Fc binding domain can block IgG Fc binding. We assessed whether IgG Fc binding to gE2/gI2 reduces ADCC titers and demonstrated significantly higher titers when cells are transfected with gD2/gI2/gE2_MUT_ DNA than with gD2/gI2/gE2_WT_DNA, where the gE2 mutation abrogates IgG Fc binding. We spiked HSV-2-seropositive sera with a gE2 mAb, B1E6, that blocks IgG Fc binding and noted a significant increase in ADCC titers, while a gE2 mAb, E1, that fails to block IgG Fc binding has no effect. These results indicate that antibodies to gE2 may counteract the inhibition of ADCC by gE2/gI2, supporting the concept that gE2 antibodies induced by a vaccine may prevent gE2/gI2-mediated immune evasion. The results also suggest that HSV-2 infection, even in subjects with many annual recurrences of genital herpes, does not produce sufficient antibodies that block the ability of gE2/gI2 to bind IgG Fc.

Several observations support that ADCC contributes to controlling HSV infection. First, in a vaccine study to prevent genital herpes, immunized individuals received gB2 and gD2 subunit proteins adjuvanted with MF-59. Immunization did not protect against genital herpes despite good neutralizing antibody titers (49). In a post-study analysis, a poor ADCC response to the vaccine was noted, and this poor response was proposed as a possible explanation for the lack of vaccine protection (50). Our current study identified that gE2/gI2 inhibits ADCC and may account for low ADCC responses to the gB2/gD2 vaccine, as we previously postulated (51). Second, newborns with high transplacental neutralizing antibody titers to HSV were protected from neonatal herpes (52). In a subsequent study, ADCC and neutralizing antibodies each contributed to protection independently (53). Third, studies in a mouse model of neonatal herpes using a single-cycle HSV-2 strain deleted in glycoprotein D (gD2) demonstrated that protection in newborn mice correlated with ADCC titers, as did studies involving antibody passive transfer (54–56). Therefore, having potent ADCC responses to a herpes vaccine is likely important.

We previously demonstrated, using the murine flank model, that a gE1 mutant virus, defective in IgG Fc binding but intact for cell-to-cell spread, which is another function mediated by gE1, was more than 100-fold less virulent than the wild-type virus. To circumvent the observation that the Fc domain of mouse IgG does not bind to gE1 or gE1/gI1, we passively immunized the mice with human anti-HSV IgG (46). We determined that gE1 contributed to virulence by inhibiting ADCC and antibody-dependent complement activation (45, 57). Here, we demonstrated that full-length gE2, when co-transfected with full-length gI2, binds the IgG Fc domain of human IgG to inhibit NK cell activation, a biomarker for ADCC. Wild-type gE2 co-transfected with gI2 reduced ADCC titers by 6.5-fold compared with a gE2 mutant co-transfected with gI2, which was incapable of binding anti-HSV-2 IgG Fc.

A potential limitation of the current study is that we did not measure ADCC responses to the HSV-2 trivalent gC2, gD2, gE2 mRNA vaccine using sera collected from immunized mice or guinea pigs (36, 58). Assessing ADCC using mouse or guinea pig vaccine sera requires using NK cells with Fcγ receptors that bind murine or guinea pig IgG Fc. The human FcγRIIIA receptor on NK-92 cells does not bind well to mouse IgG Fc or, in our experience, to guinea pig IgG Fc (48). Our goals were to determine whether gC2, gD2, and gE2 are targets of human antibodies that mediate ADCC; to assess whether gE2/gI2 inhibits ADCC; and to demonstrate that a gE2 mAb can prevent this inhibition and restore ADCC titers. We addressed each of these goals in the current study. Future studies can use mouse or guinea pig NK cells to assess the importance of ADCC responses in sera from immunized or infected animals. However, *in vivo* studies in mice and guinea pigs may falsely inflate the importance of ADCC because gE2/gI2 does not bind the IgG Fc domain of mouse or guinea pig IgG (28). *In vivo* studies to address the contribution of gE2/gI2 to immune evasion will require passive transfer of human IgG, as we did for HSV-1, or passive transfer of mAbs with humanized IgG Fc domains (25).

Many human pathogens, including herpesviruses and bacteria, encode IgG Fc-binding proteins. These pathogens include HSV-1 (gE1, gE1/gI1), HSV-2 (gE2/gI2), HCMV (gp34, gp68), and VZV (gE), *Staphylococcus aureus* (protein A), *Streptococcus pyogenes* (protein G), and *Peptostreptococcus* (protein L) (28, 45, 59–63). The IgG Fc domain mediates important host defenses in addition to ADCC, including complement activation and antibody-dependent phagocytosis (64). If immunization aimed at blocking IgG Fc binding to HSV blocks these functions and is effective as part of a vaccine strategy, the approach may apply to many pathogens.

## Data Availability

All the data in this study can be found in the text, figures, and **Supplementary Materials**.
