# Supplementary material for: HSV-2 gE2/gI2 are immune evasion molecules that bind IgG Fc to inhibit antibody-dependent cellular cytotoxicity
Source: Front Immunol. 2026 Mar 3;17:1766722. doi: 10.3389/fimmu.2026.1766722 (PMC13011038; doi:10.3389/fimmu.2026.1766722)
Supplement: Supplementary file 1 [file Table1.docx]

**Supplementary Table 1**

Suppl. Table 1. Positive and negative controls for NK cell CD107a surface expression

| Figure number | NK CD107a expression | | | SKOV3 cells | | |
| --- | --- | --- | --- | --- | --- | --- |
|  | Sample size | PMA (pos. control) | No PMA (neg. control) | Sample size | Herceptin (pos. control) | No Herceptin (neg. control) |
| 2B | n=7 | 83.8% ± 2.2% | 0.38% ± 0.04 | n=7 | 25.0% ± 3.0 | 1.32% ± 0.36 |
| 2D | n=1 | 81.0% | 0.24% | n=3 | 33.3% ± 0.1 | 2.16% ± 0.18 |
| 4C | n=6 | 87.2% ± 3.0 | 0.29% ± 0.05 | n=6 | 28.0% ± 2.3 | 1.03% ± 0.23 |
| 4E | n=2 | 78.0% ± 3.3 | 0.20% ± 0.01 | n=3 | 24.1% ± 4.5 | 0.46% ± 0.07 |
| 6B | n=3 | 83.1% ± 5.0 | 0.36% ± 0.05 | n=3 | 28.6% ± 8.0 | 1.47% ± 0.62 |
| 6D | n=2 | 77.0% ± 3.6 | 0.50% ± 0.02 | n=2 | 27.3 % ± 2.7 | 0.57 % ± 0.13 |

The table lists the results of positive and negative controls used for NK cell CD107a surface expression for assays shown in Figures 2, 4, and 6. PMA was used to stimulate CD107a expression in the absence of antibodies and target cells. Herceptin is an antibody that binds to HER2 receptors on SKOV3 cells, serving as a positive control for NK cell CD107a expression. ±, represents SEM.

**Supplementary Figure 1.**

1. **HSV-2 gE2 strain 2.12 DNA and amino acid sequence**

gE2 and gI2 2.12 DNA and amino acid sequences

gE2 DNA sequence

ATGGCTCGCGGGGCCGGGTTGGTGTTTTTTGTTGGAGTTTGGGTCGTATCGTGCCTGGCGGCAGCACCCAGAACGTCCTGGAAACGGGTAACCTCGGGCGAGGACGTGGTGTTGCTTCCGGCGCCCGCGGGGCCGGAGGAACGCACCCGGGCCCACAAACTACTGTGGGCCGCGGAACCCCTGGATGCCTGCGGTCCCCTGCGCCCGTCGTGGGTGGCGCTGTGGCCCCCCCGACGGGTGCTCGAGACGGTCGTGGATGCGGCGTGCATGCGCGCCCCGGAACCGCTCGCCATAGCATACAGTCCCCCGTTCCCCGCGGGCGACGAGGGACTGTATTCGGAGTTGGCGTGGCGCGATCGCGTAGCCGTGGTCAACGAGAGTCTGGTCATCTACGGGGCCCTGGAGACGGACAGCGGTCTGTACACCCTGTCCGTGGTCGGCCTAAGCGACGAGGCGCGCCAAGTGGCGTCGGTGGTTCTGGTCGTGGAGCCCGCCCCTGTGCCGACCCCGACCCCCGACGACTACGACGAAGAAGACGACGCGGGCGTGAGCGAACGCACGCCGGTCAGCGTTCCCCCCCCAACCCCCCCCCGTCGTCCCCCCGTCGCCCCCCCGACGCACCCTCGTGTTATCCCCGAGGTGTCCCACGTGCGCGGGGTAACGGTCCATATGGAGACCCCGGAGGCCATTCTGTTTGCCCCCGGGGAGACGTTTGGGACGAACGTCTCCATCCACGCCATTGCCCACGACGACGGTCCGTACGCCATGGACGTCGTCTGGATGCGGTTTGACGTGCCGTCCTCGTGCGCCGAGATGCGGATCTACGAAGCTTGTCTGTATCACCCGCAGCTTCCAGAGTGTCTATCTCCGGCCGACGCGCCGTGCGCCGTAAGTTCCTGGGCGTACCGCCTGGCGGTCCGCAGCTACGCCGGCTGTTCCAGGACTACGCCCCCGCCGCGATGTTTTGCCGAGGCTCGCATGGAACCGGTCCCGGGGTTGGCGTGGCTGGCCTCCACCGTCAATCTGGAATTCCAGCACGCCTCCCCCCAGCACGCCGGCCTCTACCTGTGCGTGGTGTACGTGGACGATCATATCCACGCCTGGGGCCACATGACCATCAGCACCGCGGCGCAGTACCGGAACGCGGTGGTGGAACAGCACCTCCCCCAGCGCCAGCCCGAGCCCGTCGAGCCCACCCGCCCGCACGTGAGAGCCCCCCCTCCCGCGCCCTCCGCGCGCGGCCCGCTGCGCCTCGGGGCGGTGCTGGGGGCGGCCCTGTTGCTGGCCGCCCTCGGGCTGTCCGCGTGGGCGTGCATGACCTGCTGGCGCAGGCGCTCCTGGCGGGCGGTTAAAAGCCGGGCCTCGGCGACGGGCCCCACTTACATTCGCGTGGCGGACAGCGAGCTGTACGCGGACTGGAGTTCGGACAGCGAGGGGGAGCGCGACGGGTCCCTGTGGCAGGACCCTCCGGAGAGACCCGACTCTCCCTCCACAAATGGATCCGGCTTTGAGATCTTATCACCAACGGCTCCGTCTGTATACCCCCATAGCGAGGGGCGTAAATCTCGCCGCCCGCTCACCACCTTTGGTTCGGGAAGCCCGGGCCGTCGTCACTCCCAGGCCTCCTATTCGTCCGTCCTCTGGTAA

gE2 2.12 amino acid sequence

MARGAGLVFFVGVWVVSCLAAAPRTSWKRVTSGEDVVLLPAPAGPEERTRAHKLLWAAEPLDACGPLRPSWVALWPPRRVLETVVDAACMRAPEPLAIAYSPPFPAGDEGLYSELAWRDRVAVVNESLVIYGALETDSGLYTLSVVGLSDEARQVASVVLVVEPAPVPTPTPDDYDEEDDAGVSERTPVSVPPPTPPRRPPVAPPTHPRVIPEVSHVRGVTVHMETPEAILFAPGETFGTNVSIHAIAHDDGPYAMDVVWMRFDVPSSCAEMRIYEACLYHPQLPECLSPADAPCAVSSWAYRLAVRSYAGCSRTTPPPRCFAEARMEPVPGLAWLASTVNLEFQHASPQHAGLYLCVVYVDDHIHAWGHMTISTAAQYRNAVVEQHLPQRQPEPVEPTRPHVRAPPPAPSARGPLRLGAVLGAALLLAALGLSAWACMTCWRRRSWRAVKSRASATGPTYIRVADSELYADWSSDSEGERDGSLWQDPPERPDSPSTNGSGFEILSPTAPSVYPHSEGRKSRRPLTTFGSGSPGRRHSQASYSSVLW*

gI2 DNA sequence

ATGCCCGGCCGCTCGCTGCAGGGCCTGGCGATCCTGGGCCTGTGGGTCTGCGCCACCGGCCTGGTCGTCCGCGGCCCCACGGTCAGTCTGGTCTCAGACTCACTCGTGGATGCCGGGGCCGTGGGGCCCCAGGGCTTCGTGGAAGAGGACCTGCGTGTTTTCGGGGAGCTTCATTTTGTGGGGGCCCAGGTCCCCCATACAAACTACTACGACGGCATCATCGAGCTGTTTCACTACCCCCTGGGGAACCACTGCCCCCGCGTTGTACACGTGGTCACACTGACCGCATGCCCCCGCCGCCCCGCCGTGGCGTTCACCTTGTGTCGCTCGACGCACCACGCCCACAGCCCCGCCTATCCGACCCTGGAGCTGGGTCTGGCGCGGCAGCCGCTTCTGCGGGTTCGAACGGCAACGCGCGACTATGCCGGTCTGTATGTCCTGCGCGTATGGGTCGGCAGCGCGACGAACGCCAGCCGGTTTGTTTTGGGGGTGGCGCTCTCTGCCAACGGGACGTTTGTGTATAACGGCTCGGACTACGGCTCCTGCGATCCGGCGCAGCTTCCCTTTTCGGCCCCGCGCCTGGGACCCTCGAGCGTATACACCCCCGGAGCCTCCCGACCCACCCCTCCACGGACAACGACACCCCCGTCCTCCCCCCGAGACCCGACCCCCGCCCCCGGGGACACAGGGACGCCCGCGCCCGCGAGCGGCGAGATAGCCCCGCCCAATTCCACGCGATCGGCCAGCGAATCGAGACACAGGCTAACCGTAGCCCAGGTAATCCAGATCGCCATACCGGCGTCCATCATCGCCTTTGTGTTTCTGGGCAGCTGTATCTGCTTCATCCATAGATGCCAGCGCCGATACAGGCGCCCCCGCGGCCAGATTTACAACCCCGGGGGCGTTTCCTGCGCGGTCAACGAGGCGGCCATGGCCCGCCTCGGAGCCGAGCTGCGATCCCACCCAAACACCCCCCCCAAACCCCGACGCCGTTCGTCGTCGTCCACGACCATGCCTTCCCTAACGTCGATAGCTGAGGAATCGGAGCCAGGTCCAGTCGTGCTGCTGTCCGTCAGTCCTCGGCCCCGCAGTGGCCCGACGGCCCCCCAAGAGGTCTAG

gI2 amino acid sequence

MPGRSLQGLAILGLWVCATGLVVRGPTVSLVSDSLVDAGAVGPQGFVEEDLRVFGELHFVGAQVPHTNYYDGIIELFHYPLGNHCPRVVHVVTLTACPRRPAVAFTLCRSTHHAHSPAYPTLELGLARQPLLRVRTATRDYAGLYVLRVWVGSATNASRFVLGVALSANGTFVYNGSDYGSCDPAQLPFSAPRLGPSSVYTPGASRPTPPRTTTPPSSPRDPTPAPGDTGTPAPASGEIAPPNSTRSASESRHRLTVAQVIQIAIPASIIAFVFLGSCICFIHRCQRRYRRPRGQIYNPGGVSCAVNEAAMARLGAELRSHPNTPPKPRRRSSSSTTMPSLTSIAEESEPGPVVLLSVSPRPRSGPTAPQEV*

1. **Sites of mutations in the HSV-2 gE2 strain 2.12 amino acid sequence**

MARGAGLVFFVGVWVVSCLAAAPRTSWKRVTSGEDVVLLPAPAGPEERTRAHKLLWAAEPLDACGPLRPSWVALWPPRRVLETVVDAACMRAPEPLAIAYSPPFPAGDEGLYSELAWRDRVAVVNESLVIYGALETDSGLYTLSVVGLSDEARQVASVVLVVEPAPVPTPTPDDYDEEDDAGVSERTPVSVPPPTPPRRPPVAPPTHPRVIPEVSHVRGVTVHMETPEAILFAPGETFGTNVSIHAIAHDDGPYAMDVVWMRFDVPSSCAEMRIYEACLYHPQLPECLSPADAPCAVSSWAYRLAVRSYAGCSRTTPPPRCFAEARMEPVPGLAWLASTVNLEFQHASPQHAGLYLCVVYVDDHIHAWGHMTISTAAQYRNAVVEQHLPQRQPEPVEPTRPHVRAPPPAPSARGPLRLGAVLGAALLLAALGLSAWACMTCWRRRSWRAVKSRASATGPTYIRVADSELYADWSSDSEGERDGSLWQDPPERPDSPSTNGSGFEILSPTAPSVYPHSEGRKSRRPLTTFGSGSPGRRHSQASYSSVLW*

**Supplementary Fig. 1B Legend**. The color code in amino acid sequences indicates the location of amino acid changes in gE2 to form gE2 mutant strains. Mutant #1 inserts ARAA after A337; Mutant #2 replaces H245 with a G, and P317 with a G; Mutant #3 inserts ALAG after R262 (1, 2).

**Supplementary Fig. 2**

**
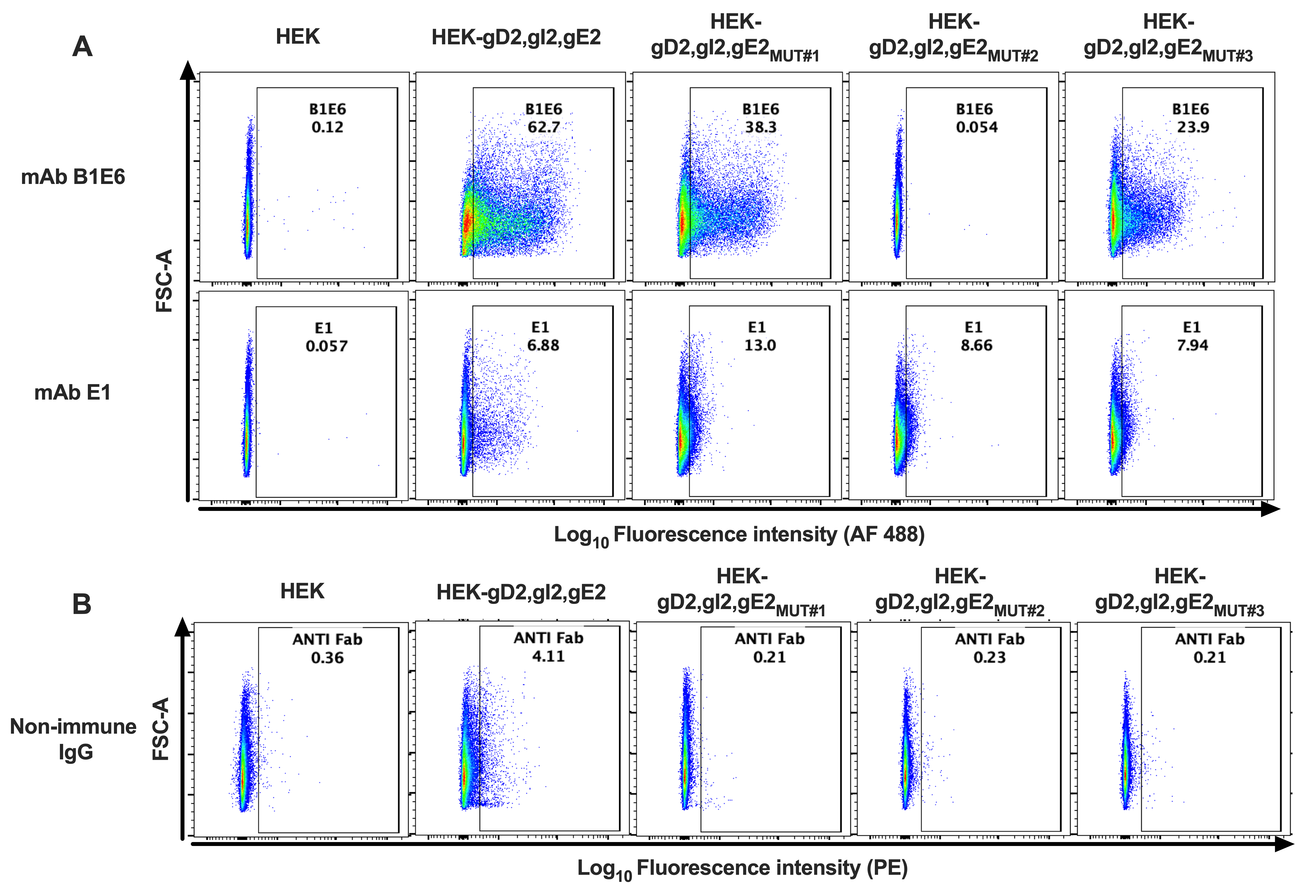
**

**Supplementary Figure 2.** **gE2_MUT_ proteins fail to bind IgG Fc**. **A.** HEK cells were transfected with gD2, gI2 and gE2_WT_ DNA or gD2, gI2 and gE2_MUT#1_, gE2_MUT#2_, or gE2_MUT#3_ DNA and evaluated for cell surface expression of gE2 by flow cytometry using two gE2 monoclonal antibodies, mAb B1E6 (top row) or E1 (bottom row), that bind to different gE2 epitopes. Both mAbs bound to gE2_WT_. E1 bound to each of the gE2_MUT_ proteins, while B1E6 failed to bind to gE2_MUT#2_, suggesting that B1E6 recognizes an epitope in gE2_WT_ that is mutated in gE2_MUT#2_. **B.** Cells expressing each of the three gE2_MUT_ proteins are defective in IgG Fc binding. HEK cells were transfected with gD2, gI2, and gE2_WT_ DNA or gE2_MUT#1_, gE2_MUT#2_, or gE2_MUT#3_ DNA, and 24 h later, cells were incubated with non-immune human IgG. Bound IgG was detected by flow cytometry using an anti-human Fab fluorophore. Non-immune human IgG bound to cells transfected with gE2_WT_ but not to cells transfected with any of the gE2_MUT_ constructs.

1. Galli JD, Horton M, Durr E, Heidecker GJ, Freed D, Fridman A, et al. Evaluation of HSV-2 gE Binding to IgG-Fc and Application for Vaccine Development. *Vaccines.* 2022;10(2):184.

2. Dubin G, Basu S, Mallory DL, Basu M, Tal-Singer R, and Friedman HM. Characterization of domains of herpes simplex virus type 1 glycoprotein E involved in Fc binding activity for immunoglobulin G aggregates. *Journal of Virology.* 1994;68(4):2478-85.
